# Abnormalities of age-related T cell senescence in Parkinson’s disease

**DOI:** 10.1186/s12974-018-1206-5

**Published:** 2018-05-28

**Authors:** C. H. Williams-Gray, R. S. Wijeyekoon, K. M. Scott, S. Hayat, R. A. Barker, J. L. Jones

**Affiliations:** 1 John Van Geest Centre for Brain Repair, Department of Clinical Neurosciences, University of Cambridge, Forvie Site, Cambridge, CB2 0PY UK; 2 Neurology Unit, Department of Clinical Neurosciences, University of Cambridge, Cambridge, UK

**Keywords:** Parkinson’s disease, Immune markers, T cells, Immunosenescence

## Abstract

**Background:**

A wealth of evidence implicates both central and peripheral immune changes as contributing to the pathogenesis of Parkinson’s disease (PD). It is critical to better understand this aspect of PD given that it is a tractable target for disease-modifying therapy. Age-related changes are known to occur in the immune system (immunosenescence) and might be of particular relevance in PD given that its prevalence rises with increasing age. We therefore sought to investigate this with respect to T cell replicative senescence, a key immune component of human ageing.

**Methods:**

Peripheral blood mononuclear cells were extracted from blood samples from 41 patients with mild PD (Hoehn and Yahr stages 1–2, mean (SD) disease duration 4.3 (1.2) years) and 41 age- and gender-matched controls. Immunophenotyping was performed with flow cytometry using markers of T lymphocyte activation and senescence (CD3, CD4, CD8, HLA-DR, CD38, CD28, CCR7, CD45RA, CD57, CD31). Cytomegalovirus (CMV) serology was measured given its proposed relevance in driving T cell senescence.

**Results:**

Markers of replicative senescence in the CD8+ population were strikingly reduced in PD cases versus controls (reduced CD57 expression (*p* = 0.005), reduced percentage of ‘late differentiated’ CD57_lo_CD28_hi_ cells (*p* = 0.007) and ‘TEMRA’ cells (*p* = 0.042)), whilst expression of activation markers (CD28) was increased (*p* = 0.005). This was not driven by differences in CMV seropositivity. No significant changes were observed in the CD4 population.

**Conclusions:**

This study demonstrates for the first time that the peripheral immune profile in PD is distinctly atypical for an older population, with a lack of the CD8+ T cell replicative senescence which characterises normal ageing. This suggests that ‘abnormal’ immune ageing may contribute to the development of PD, and markers of T cell senescence warrant further investigation as potential biomarkers in this condition.

## Background

There is abundant evidence implicating a role for inflammation and immune activation in Parkinson’s disease. Neuroinflammatory changes including microglial activation and increased concentrations of pro-inflammatory cytokines are well described in PD postmortem brain [1, 2], and microglial activation can be observed in vivo even in the early stages of the disease using [11C]PK-11195 PET neuroimaging [3–5]. Inflammatory cytokines including IL-6 and IL-8 are elevated in the cerebrospinal fluid (CSF) in PD compared in controls [6, 7], and immune alterations have been reported in peripheral blood including changes in monocyte [8] and lymphocyte subsets in PD [9, 10], which may be driven by a specific immune response to alpha synuclein [11]. Whilst such immune and inflammatory changes in PD might be, at least in part, secondary phenomena, immune manipulation in animal models of PD has been shown to alter disease susceptibility and severity [12, 13], suggesting that immune changes may also be a primary contributing factor to disease development. This is supported by the now well-established genetic association between the human leucocyte antigen (HLA) locus, the key genetic susceptibility factor in disorders involving pathogenic adaptive immune responses, and PD risk [14, 15]. Furthermore, our own recent data has shown that a pro-inflammatory cytokine profile in the serum in early PD has prognostic significance, being associated with more cognitive dysfunction and faster motor progression over 36 months of prospective follow-up in a large incident PD cohort [16].

Given that PD is a condition which becomes increasingly prevalent with age, the relevance of age-related immune dysregulation (known as immunosenescence) in this disorder warrants further investigation. One of the hallmarks of immunosenescence is the loss of naïve T cells, especially CD8+ cells, and the reciprocal accumulation of memory cells, particularly terminally differentiated effector CD8+ cells (TEMRAs), as a result of age-related thymic involution and repeated exposure to pathogens [17–19]. In vitro, progression of CD8 T cells to a senescent state is characterised by the loss of surface expression of the co-stimulatory molecule CD28 and expression of CD57 [17, 20]. In vivo, both TEMRA and CD28_lo_CD57_hi_ CD8+ cells are increased in frequency in patients with chronic infections, e.g. HIV, as well as in older age [21–23]. Indeed, accumulation of late differentiated CD8 cells has been proposed to be a major characteristic of an ‘immune risk profile’ in patents > 85 years which is associated with higher mortality [18, 24]. Infection with cytomegalovirus (CMV) has been proposed as the key pathogenic driver of this senescent CD8 subset [18], with T cell receptor (TCR) restricted, CMV-specific T cells comprising a large proportion of the CD8 T cells in the elderly [25, 26]. Within the CD4 population, similar phenotypic changes occur with repeated antigenic stimulation, but late differentiated CD4 cells accumulate at a much lower frequency [27]. Loss of thymic T cell production can also be measured in the CD4 pool as a loss of CD4+ recent thymic emigrants (RTEs), CD4+ naïve cells that co-express CD31 [19].

The functional impact of CD8+ T cell replicative senescence is an overall impairment of the adaptive immune response, with a consequent reduction in the ability to fight novel infections and impairment in responses to vaccinations in the elderly [17]. The impact of CD8+ cell senescence on the development and progression of PD is unknown. Such changes may reduce responses to disease-related antigens such as misfolded alpha synuclein, thus limiting clearance of toxic proteins and potentially contributing to PD. Alternatively, they may be protective through reducing the capacity of the immune system to become activated, hence limiting an important driver of central neuroinflammation [28]. Here, we sought to answer this question by investigating whether there was an association between an immunosenescent T cell phenotype and PD and to determine the relevance of CMV infection to this.

## Methods

### Subjects

Patients with early-stage PD (Hoehn and Yahr stage ≤ 2, no dementia) were recruited from the Parkinson’s Disease Research Clinic at the John Van Geest Centre for Brain Repair in Cambridge. All patients met the UKPDS Brain Bank criteria for the diagnosis of PD [29] and were aged between 55 and 80. Controls without neurological disease were recruited from the NIHR Cambridge BioResource. MAPT (microtubule-associated protein tau) H1/H2 genotypes were available for all subjects. Controls were age-, sex- and MAPT genotype-matched to patients. Exclusion criteria included current infections, chronic inflammatory or autoimmune disorders, vaccination within the past 3 weeks, surgery within the past month, and recent use of immunosuppressive therapy (< 1 year), oral or intravenous steroids (< 3 months), non-steroidal anti-inflammatories (< 2 weeks) or aspirin > 75 mg (< 2 weeks). Control subjects had no history of neurological disease, no self-reported memory problems and no depression. PD cases underwent full clinical assessment including medical history and comorbidities, the Movement Disorder Society-Unified Parkinson’s Disease Rating Scale (MDS-UPDRS), Hoehn and Yahr Stage, Addenbrooke’s Cognitive-Examination-Revised (ACE-R), assessment of semantic fluency (animal naming in 90 s) and pentagon copying and the Beck Depression Inventory. Levodopa equivalent daily doses were calculated [30]. Cases were stratified in terms of prognosis using factors identified in our previous CamPaIGN study: they were classified as high dementia risk if they carried the MAPT (microtubule-associated protein tau) H1/H1 genotype and performed poorly on tests of semantic fluency (< 20 animals in 90 s) and/or pentagon copying (< 2/2), intermediate risk if they carried one of these factors and low risk if they carried no risk factors [31].

### Sample processing

Up to 50 ml of venous blood was collected from each participant in a combination of lithium heparin tubes for peripheral blood mononuclear cell (PBMC) isolation, plain tubes for serum separation and ethylenediaminetetraacetic acid (EDTA) tubes for measurement of full blood count and differential count. Samples for patient-control pairs were collected on the same day (9–11 am) and processed in parallel to minimise inter-assay variability. Plain tubes were left to clot for 15 min before centrifugation at 2000 rpm and removal of serum, which was stored at − 80 °C prior to subsequent analysis for CMV IgG using a LIAISON® CMV IgG II chemiluminescent immunoassay (DiaSorin, Italy). PBMCs were separated by centrifugation over a Ficoll gradient and washed and divided into aliquots for immunophenotyping and cell separation into T cell subsets.

PBMCs for phenotyping were blocked with 2% mouse serum prior to labelling with two panels of fluorochrome-conjugated monoclonal antibodies to T cell surface markers of interest [panel 1: CD3(FITC), CD4(PECy5), CD8(APCCy7), HLA-DR(V500), CD38(PE); panel 2: CD3(APC), CD4(V500), CD8(APCCy7), CD28(PECy7), CCR7(FITC), CD45RA(PECy5), CD57(PE), CD31(V450), BD Biosciences). Cells were fixed with 2% paraformaldehyde. Flow cytometry was performed on a BD Biosciences FACS CantoII with BD FACS Diva software. Isotype-matched mouse monoclonal antibodies were used where appropriate to exclude non-specific antibody binding. Unstained lymphocytes were also used as negative controls.

### Data analysis

Immunophenotyping data was analysed using Flowjo. Lymphocytes were gated using forward scatter and side scatter, and single cells were identified using forward-scatter area versus width. CD4 and CD8 subsets were identified by gating on CD3-high CD4-high and CD3-high CD8-high cells respectively. Naïve, central memory, effector memory and terminally differentiated memory CD45RA+ cells (TEMRA) were identified based on CCR7 and CD45RA expression [19]. Senescent ‘late differentiated’ cells were defined as CD28-low CD57-high cells within the CD8 population. RTEs were identified as CD31+ cells within the CD4 naïve (CCR7+CD45RA+) population. Expression of markers of activation (CD28, CD38, HLA-DR) and senescence (CD57) were quantified within CD4+ and CD8+ populations as the ratio of median fluorescence intensity in labelled versus unlabelled lymphocytes (MFI ratio), to allow for variations in voltage between samples.

The primary statistical analyses involved comparison of T cell subsets (as a percentage of lymphocytes) and MFI ratios for relevant markers in PD cases versus matched controls using paired *t* tests. CMV positivity in patients versus controls was compared using chi-square tests, and analyses of variance (ANOVA), including age as a covariate, were used for patient-control comparisons of T cell markers in CMV-positive and CMV-negative subgroups. Relationships between relevant markers and clinical measures of motor and cognitive functions were explored using Pearson’s correlations. Statistical analysis was performed using GraphPad Prism version 6.0 and SPSS version 25 (IBM).

## Results

Forty-one patients with PD and 41 age/gender-matched controls were recruited. Demographic and clinical characteristics of the subjects and CMV status are shown in Table 1. Nine PD cases were designated high dementia risk, 18 were low risk and 14 were intermediate risk. Analysis of full blood and differential counts in *n* = 20 PD and 20 controls revealed lower total lymphocyte counts in PD cases than controls (1.4 (0.4) versus 1.9 (0.6) × 10^9^/L; *p* = 0.01) with no significant differences in monocyte or neutrophil numbers. Flow cytometric data from the full cohort indicated no difference in the CD4:CD8 ratio between PD cases and controls [3.8 (2.3) versus 3.3 (3.0); *p* > 0.05].

**Table 1:** Demographic and clinical characteristics of subjects

Variable	PD (*n* = 41)	Controls (*n* = 41)	*p*
Age (years)	68.4 (6.3)	68.1 (5.6)	0.85
Gender (% male)	68.3	68.3	1.0
Disease duration (years)	4.3 (1.2)		
MDS-UPDRS motor score	34.9 (12.4)		
ACE-R	93.0 (8.3)		
Levodopa equivalent daily dose (mg)	591.5 (292.9)		
CMV IgG (% positive)	46.3	61.0	0.18

Values shown are mean (SD) unless otherwise stated. Continuous variables compared using Student’s *t* tests and categorical variables compared using chi-square tests or Fisher’s exact test as appropriate

*MDS-UPDRS* Movement Disorder Society Unified Parkinson’s Disease Rating Scale, *ACE-R* Addenbrooke’s Cognitive Examination-Revised

**Fig. 1:**
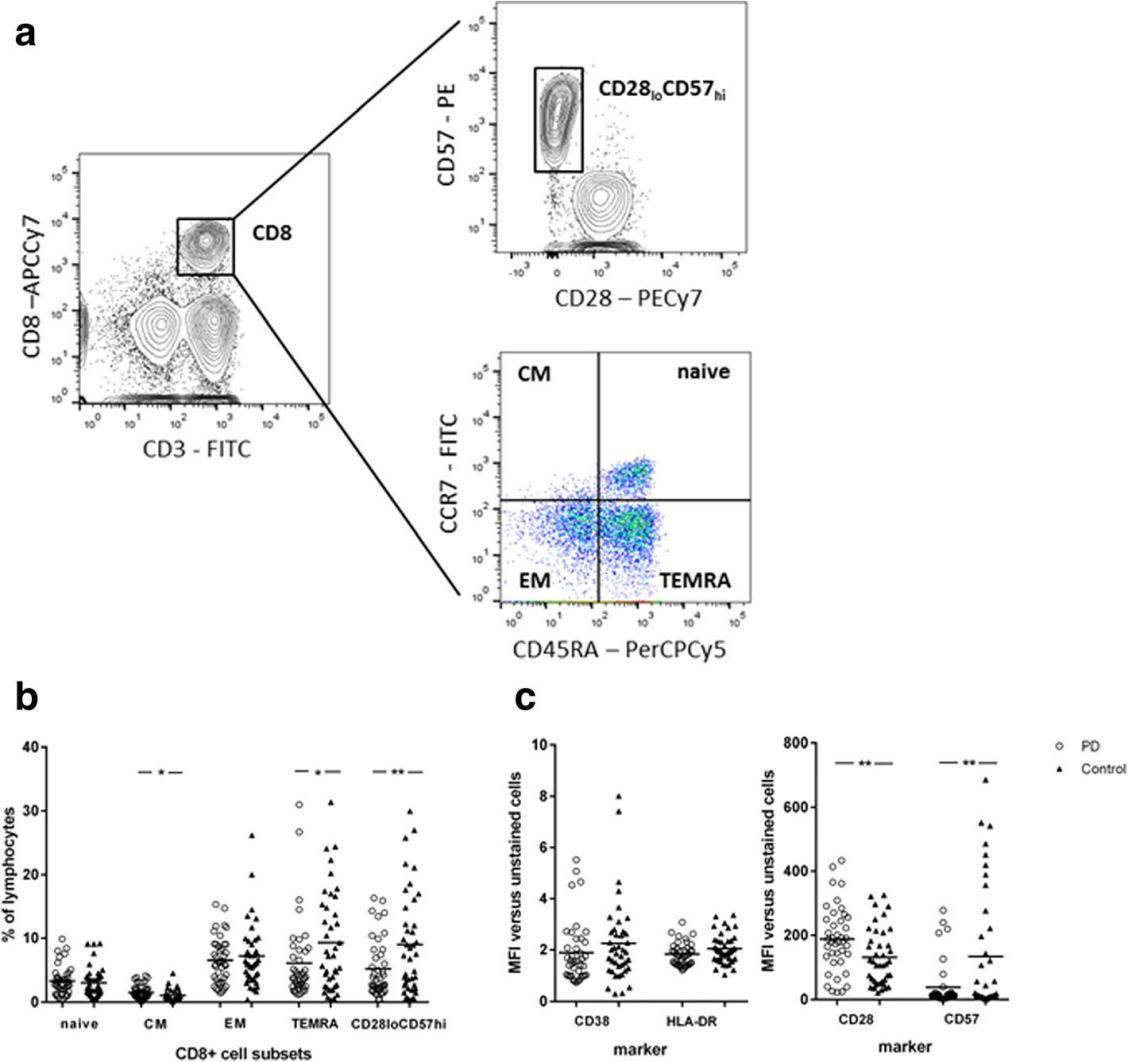
CD8 immunophenotyping in PD cases (*n* = 41) versus age-matched controls (*n* = 41). **a** Following gating of the lymphocyte and singlet populations, CD8 cells were gated on the basis of high CD3 and CD8 staining, and the CD28_lo_CD57_hi_ subset was identified as a discrete population of CD8+ cells using 5% contour plots. Quadrant gates were used to divide the CD8 population into naïve, central memory, effector memory and TEMRA (terminally differentiated effector memory CD45RA+ve cells) subsets on pseudocolour dot plots. **b** Individual-level data for CD8 cell subsets (shown as percentage of lymphocytes). **c** Individual-level data for surface marker expression in CD8 T cells (median fluorescence intensity (MFI) ratio versus unstained lymphocytes). Horizontal lines denote means; *p < 0.05, **p < 0.01 in paired *t* tests

However, there was a reduction in the number and proportion of CD28_lo_CD57_hi_CD8+ T cells in individuals with PD compared to controls, along with a marginally significant reduction in CD8+ TEMRA cells and accompanying small increase in CD8+ central memory cells (Table 2 and Fig. 1a, b). Expression of the activation markers CD38 and HLA-DR on CD8+ T cells was not different between patients and controls, but expression of CD57 was reduced and expression of CD28 was increased in PD patients (Table 3; Fig. 1c), in keeping with the CD8+ subset data (Table 3; Fig. 1c). No differences were identified in the CD4+ T cell pool between patients and controls.

**Table 2:** T lymphocyte subsets

	Subset (% of lymphocytes)	PD	Control	*p*
CD8	Naïve	3.4 (2.3)	3.1 (2.4)	0.511
	Central memory	1.5 (1.2)	1.1 (0.9)	0.015
	Effector memory	6.6 (3.6)	7.2 (5.0)	0.516
	TEMRA	6.2 (6.3)	9.3 (7.9)	0.042
	CD28_lo_57_hi_	5.3 (4.5)	9.0 (8.0)	0.007*
CD4	Naïve	17.5 (9.0)	16.0 (8.2)	0.406
	Central memory	18.0 (6.7)	15.8 (6.7)	0.052
	Effector memory	14.2 (6.5)	12.8 (6.4)	0.245
	TEMRA	2.1 (2.4)	2.0 (2.3)	0.747
	RTE	2.2 (3.3)	2.1 (2.2)	0.827

T lymphocyte subsets are expressed as a percentage of the lymphocyte population.

*TEMRA* terminally differentiated effector memory CD45RA+ve cells, *RTE* recent thymic emigrants

*p value (from paired *t* test) which remains < 0.05 following Bonferroni correction for multiple testing

**Table 3:** T cell surface marker expression

	Marker	PD	Control	*p*
CD8	CD28	188.6 (102.5)	131.5 (88.3)	0.005*
	CD38	1.9 (1.2)	2.3 (1.6)	0.117
	HLA-DR	1.8 (0.4)	2.0 (0.6)	0.055
	CD57	38.2 (69.9)	161.0 (261.7)	0.005*
CD4	CD28	334.6 (97.3)	299.8 (89.1)	0.067
	CD38	6.1 (3.0)	7.1 (4.0)	0.057
	HLA-DR	1.1 (0.2)	1.2 (0.2)	0.413
	CD57	1.8 (1.5)	2.0 (1.3)	0.493

Values shown are mean (SD) ratio of median fluorescent intensity versus unstained lymphocytes (MFI ratio)

*p value which remains < 0.05 following Bonferroni correction for multiple testing

For cell subsets/markers reaching significance (*p* < 0.05), ANOVA were performed to assess the effect of dementia risk group on the observed case-control differences (with ‘case-control status’ and ‘risk subgroup’ included as fixed factors and age and gender as covariates). Main effects of ‘case-control status’ were confirmed for the markers previously identified, but there was no interaction with risk subgroup. Amongst the PD cases, no significant correlations were found between T cell subset percentages, or surface markers of activation and senescence, and either clinical measures of motor and cognitive function or equivalent daily levodopa dose.

**Fig. 2:**
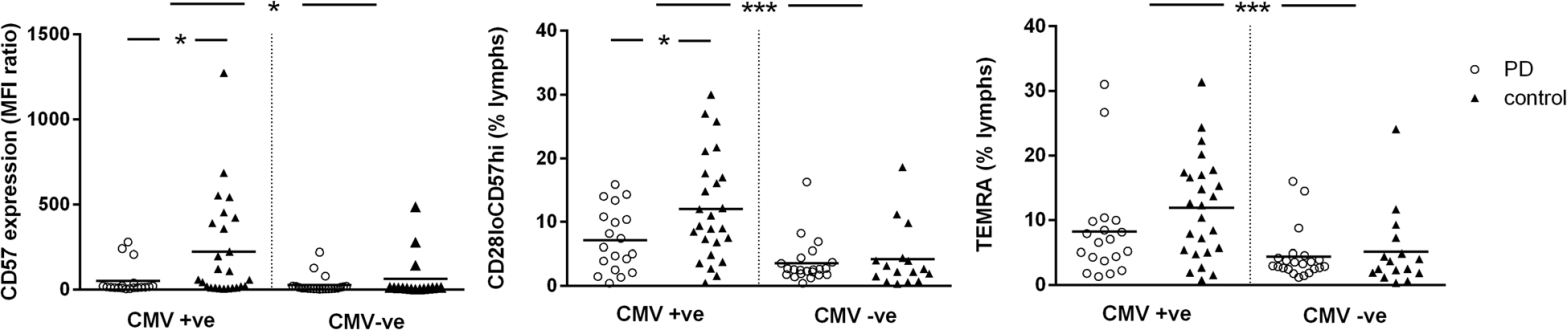
CD8+ senescence markers in CMV-positive versus CMV-negative subjects. The figure shows CD57 expression on CD8+ lymphocytes (median fluorescence intensity (MFI) ratio versus unstained lymphocytes), CD8+ CD28_lo_CD57_hi_ cells (percentage of lymphocytes) and TEMRA cells (percentage of lymphocytes) in PD versus age-matched controls stratified by CMV seropositivity. Horizontal lines denote means; *p < 0.05 and ***p ≤ 0.001

CMV IgG seropositivity was not significantly different between PD cases (19/41) and controls (25/41) (*p* = 0.18). Nonetheless, given the previously described association between CMV exposure and CD8 immunosenescence, we explored this relationship further. As anticipated, CD8+ senescence markers were elevated in CMV-positive versus CMV-negative subjects overall, including CD57 expression (ANOVA with age as covariate, *F* = 4.66, *p* = 0.03), CD28_lo_CD57_hi_ cells (% of lymphocytes, *F* = 18.75, *p* < 0.001) and TEMRA cells (% of lymphocytes, *F* = 12.71, *p* = 0.001). However, this effect was more apparent for controls than for PD patients, with significantly higher CD57 expression (*p* = 0.017) and CD28_lo_CD57_hi_ cells (% of lymphocytes, *p* = 0.028) in controls versus PD cases in the CMV-positive group (Fig. 2).

## Discussion

This study demonstrates for the first time that the peripheral immune profile in PD is distinctly atypical for a more elderly population, with a lack of the CD8+ T cell replicative senescence which characterises normal ageing. Although other authors have reported phenotypic alterations in the T lymphocyte population in PD, these alterations have mainly been restricted to CD4+ cell subsets with conflicting findings [9, 32, 33] and we found no changes in the CD4+ T cell pool in this study. No previous studies have specifically explored markers of T cell replicative senescence in PD. CMV infection has been strongly implicated in CD8+ replicative senescence [18], but this does not appear to be confounding our observations, with similar levels of CMV IgG positivity in both patient and control groups. Instead, the senescent shift typically induced by chronic latent CMV infection is not observed in this PD cohort. This is an intriguing finding, raising the possibility that there are inherent differences in the adaptive immune response to chronic viral infections in PD. We hypothesise that these differences may contribute to a more active T cell-mediated immune response to PD-relevant antigens such as alpha synuclein, leading in turn to an exaggerated neuroinflammatory response which may contribute to the neuropathology of PD.

In keeping with this hypothesis, there is well-established evidence from genetic studies of an immune-mediated susceptibility to PD, with association between the HLA locus, which is critical in antigen recognition, and PD risk [14, 34], as well as more widespread genomic associations in loci affecting T cell regulation and function [35, 36]. Furthermore, specific T cell responses to epitopes of alpha synuclein have recently been described at a higher frequency in PD patients than controls by Sulzer and colleagues [11] and were closely associated with possession of known PD risk alleles at the HLA locus. In addition, alpha synuclein peptides have been shown to bind to these HLA ‘risk variant’ molecules in vitro, thus revealing a potential functional mechanism through which the well-described HLA association may contribute to the development of PD. Whilst the authors of this work highlighted the importance of the Th2-type CD4+ cell-mediated response observed in their experiments, they also found evidence of a significant CD8+-mediated response when stimulating cells with shorter alpha synuclein peptides, and in fact, the strongest association between alpha synuclein-specific T cell responses and HLA was seen for the MHC class I allele HLA A*11.02, thus underlining the relevance of the CD8 (MHC class 1 restricted) response [11]. The latter would be in keeping with our observations that PD-associated immunophenotypic changes in our cohort were within the CD8+ population. Other authors have similarly shown an elevated CD8+ T cell response to alpha synuclein peptides and herpes simplex virus peptides in PD versus controls [37].

The mechanism by which alterations in peripheral CD8+ T cell function and CNS pathology interact in PD is still not fully elucidated. However, it is now clear that there is an abundance of T lymphocytes within the meninges which keep the brain under immune surveillance and which drain, together with CNS antigens, via the CNS lymphatic system to the local lymph nodes [38]. These meningeal T cells may be activated by ‘alarm signals’ released into the CSF by degenerating CNS neurones [39] and in turn produce inflammatory cytokines which can diffuse into the brain parenchyma and lead to further activation of already ‘primed’ microglia [40] and/or impact directly on neuronal function [41]. Furthermore, the presence of CD8+ cells within the brain parenchyma in postmortem tissue from PD patients raises the possibility of direct interaction with neurones presenting relevant antigen in the context of MHC class 1, with consequent cytotoxic effects [12].

In this study, we adopted a hypothesis-driven approach and so restricted our T cell phenotyping to specifically explore cell subsets and markers relevant to activation and replicative senescence. A consequent limitation of the study is that we were not able to fully characterise the T cell population in our cohort, nor assess changes in T regulatory cells, which have been reported to be reduced in both number, and functional ability to suppress effector T cells in PD patients [9]. Another potential limitation of our study is that the majority of our patients were taking dopaminergic medication, which has been implicated as a potential confounding factor in alterations in lymphocyte populations in PD cohorts [33]. In particular, in vitro data indicates that dopamine is an activator of effector T cells and an inhibitor of regulatory T cells [42]. However, studies specifically comparing medicated and unmedicated PD patients have found no relationship between levodopa treatment and changes in T cell subsets [32]. Similarly, we found no associations between T cell phenotypes or surface marker expression and equivalent levodopa dose in our patient cohort suggesting that medication was not a significant factor driving the observed reduction in markers of CD8 replicative senescence.

Although we found clear differences in T cell senescence markers between patient and control groups, we were not able to demonstrate a relationship between these markers and dementia risk status or clinical measures of disease severity within the PD group. This may be because the observed immunophenotypic differences are most relevant to the earliest stages of the disease, impacting on susceptibility but not on the rate of progression. However, the power of our study to detect differences between dementia risk subgroups was likely suboptimal given the size of our cohort. In terms of measures of disease severity, the spread across the group was relatively minimal (Table 1) which consequently limited our ability to explore correlations with disease stage. Further studies exploring these markers both across a range of disease stages, and longitudinally in individual patients, would be of interest.

## Conclusions

Our data reveal that there is a reduction in age-associated replicative senescence of CD8+ T cells in PD patients compared with strictly age-matched controls. Hence, whilst immunosenescence may be detrimental in terms of increased susceptibility to infections and cancers with increasing age, it may provide a degree of protection against age-related neurodegenerative disorders such as PD, through limiting immune activation to aberrant peptides associated with these diseases. On a practical level, this work suggests that the CD8+ CD28_lo_CD57_hi_ immunophenotype warrants further evaluation as a biomarker in PD, which could be relevant for patient stratification for clinical trials of immunomodulating therapies. Furthermore, whilst immunomodulatory strategies targeting the CD4 population are being developed [43], the CD8 population may also warrant consideration as a target for immune therapies in PD.

## Abbreviations

ACE-R: Addenbrooke’s Cognitive Examination-Revised
ANOVA: Analysis of variance
CMV: Cytomegalovirus
CSF: Cerebrospinal fluid
EDTA: Ethylenediaminetetraacetic acid
HLA: Human leucocyte antigen
MAPT: Microtubule-associated protein tau
MDS-UPDRS: Movement Disorder Society Unified Parkinson’s Disease Rating Scale
MFI: Median fluorescence intensity
PBMC: Peripheral blood mononuclear cell
PD: Parkinson’s disease
RTE: Recent thymic emigrant
SD: Standard deviation
TEMRA: Terminally differentiated effector memory CD45RA+ cells
